# Eleventh International Symposium on Recent Advances in Environmental Health Research

**DOI:** 10.3390/ijerph120707635

**Published:** 2015-07-08

**Authors:** Paul B. Tchounwou

**Affiliations:** National Institutes of Health RCMI—Center for Environmental Health, College of Science, Engineering and Technology, Jackson State University, 1400 Lynch Street, Box 18750, Jackson, MI 39217, USA; E-Mail: paul.b.tchounwou@jsums.edu; Tel.: +1-601-979-2153; Fax: +1-601-979-0570

This special issue of the *International Journal of Environmental Research and Public Health* is dedicated to the publication of selected papers presented at the *Eleventh International Symposium on Recent Advances in Environmental Health Research*. The symposium was organized by Jackson State University (JSU) from 14–18 September 2014 at the Jackson Convention Complex in Jackson, Mississippi. It was built upon the overwhelming success of ten previous symposia hosted by JSU.

The *Eleventh International Symposium on Recent Advances in Environmental Health Research* served as an appropriate venue to discuss and share the latest developments and scientific advances in biomedical, environmental health and public health. This scientific conference continues to attract biomedical, environmental health and public health scientists and engineers who are at the forefront of research and innovation in the exciting field of environmental health, and who are dedicated to providing a scientific basis for informed decision-making regarding the cost-effective control and/or prevention of environmental hazards and environmentally-induced diseases. It also offered excellent opportunities for networking, sharing ideas, and sustaining and/or establishing new research collaborations and partnerships [1,2,3,4].

The first ten symposia (2004 to 2013) provided a solid foundation for the implementation of important activities of *Eleventh International Symposium on Recent Advances in Environmental Health Research* (2014). Hence, efforts were made to articulate and discuss many essential topics that included: Emerging Topics in Computational Biology and Environmental Modeling; Environmental Toxicology and Health Risk Assessment; Health Disparities and Environmental Security; Medical Geology and Human Health; Nanoscience, Nanotechnology and Nanotoxicology, Natural Resources Damage Assessment and Management; and New Frontiers in Environmental Health Research [1,2,3,4].

As in previous years, the 11th Symposium attracted about 300 participants originating from 21 different countries. A total of 242 scientific presentations were made across the disciplines of environmental health, biomedical and clinical sciences, and public health. Original contributions were solicited on relevant symposium topics, and submitted manuscripts were subject to a rigorous peer-review process conducted according to the guidelines and high standards for publication in International Journal of Environmental Research and Public Health-IJERPH [5]. With its recent 2014-impact factor of 2.063, IJERH has continued to maintain its rank as one of the premier journals advancing research that addresses critical issues related to environmental quality and public health. This high quality journal is covered by leading indexing services including PubMed (Medline) and the Science Citation Index Expanded (Web of Science), EMBASE and Scopus (SciVerse). Full-text articles are also available through PubMed Central [5].

I wish to extend special thanks to Dr. Prescott Deininger (Director of Tulane Cancer Center and Professor at the Department of Epidemiology, Tulane University School of Public Health and Tropical Medicine, New Orleans, LA, USA), and. Dr. Stella Anyangwe (Former World Health Organization Representative, Independent Global Health Expert, and Honorary Professor, School of Health Systems and Public Health, Faculty of Health Sciences, University of Pretoria, Pretoria, South Africa), for serving as Distinguished Speakers for the Biomedical Sciences and Health Information Lecture Series that was held in conjunction with the symposium. Dr. Deininger spoke on “Mobile Elements as a Source of Environmentally Sensitive Genetic Instability” and Dr. Anyangwe presented on “Global Health and Neglected Tropical Diseases”. Other plenary presentations and keynote addresses were made by prominent biomedical and environmental health scientists and engineers with research expertise in cancer, diabetes, HIV/AIDS, infectious and parasitic diseases, cardiovascular diseases, neurodegenerative diseases, gene-environment interactions, nanoscience and nanomedicine, emerging technologies, health disparities and other environmentally-related illnesses. These important health issues were associated with the symposium topics [1,2,3,4].

I would like to commend the authors for their excellent contributions to advancing scientific research and providing the necessary tools to guide policies on environmental issues and public health protection. Special thanks are also extended to all our peer-reviewers who helped to critique the research and improve the scientific quality. To all my colleagues and staff who worked very hard to make the symposium a total success, I say “Thanks a million” for a job well done.

Special thanks are extended Dr. Carolyn W. Meyers (President), Dr. James C. Renick (Provost and Senior Vice President of Academic and Student Affairs), and Dr. Loretta Moore (Vice President for Research and Federal Relations). On behalf of the entire organizing committee, the greatest acknowledgments go to our major symposium sponsors including the National Institutes of Health (NIH) RCMI-Center for Environmental Health, the U.S. Department of Education Title III Graduate Education Program, the U.S. Environmental Protection Agency, the JSU Office of Academic Affairs, and the JSU Office of Research and Federal Relations. I also commend Mrs. Rose Foster and Mrs. Wilma Templin-Branner from Oak Ridge Institute for Science and Education, and Drs. Barbara Graham and Kenneth Ndebele from Jackson State University, for their help with the organization of the pre-symposium workshop on the *National Library of Medicine (NLM) Web-Based Resources for Environmental* *Health and Biomedical Research.* As in previous years, the major emphasis of the workshop was on training participants on how to access and retrieve important environmental health and biomedical research information from relevant NLM web-based resources.

The next symposium will be held at the Jackson Marriott Hotel (Jackson, Mississippi, USA), from 13–16 September 2015. I strongly encourage all past symposium participants, and invite new biomedical and environmental health scientists and engineers who are interested in solving global environmental and public health challenges, to join us at the next conference meeting as we celebrate the 12th anniversary of this grand event.
